# Isolation and characterization of bacteriophage against clinical isolates of AmpC beta lactamase–Producing *Klebsiella pneumoniae* from hospital wastewater

**DOI:** 10.1371/journal.pone.0315079

**Published:** 2025-02-21

**Authors:** Manal Abdel Haleem A. Abusalah, Mai Abdel Haleem A. Abusalah, Chan Yean Yean, Ismail Aziah, Abdul Rahman Zaidah

**Affiliations:** 1 Department of Medical Microbiology & Parasitology, School of Medical Sciences, Universiti Sains Malaysia, Kelantan, Malaysia; 2 Faculty of Allied Medical Sciences, Department of Medical Laboratory Scinences, Al-Ahliyya Amman University, Amman, Jordan; 3 Institute for Research in Molecular Medicine (INFORMM), Universiti Sains Malaysia, Kelantan, Malaysia; 4 Hospital Universiti Sains Malaysia, Universiti Sains Malaysia, Kelantan, Malaysia; University of Gondar College of Medicine and Health Sciences, ETHIOPIA

## Abstract

**Background:**

The increasing incidence of AmpC β-lactamase producing by *K*. *pneumoniae* has raised global alarm. Consequently, there is a crucial need for effective methods to inactivate pathogenic bacteria and mitigate the associated risks. Bacteriophage therapy has been demonstrated to be an effective and alternative approach for targeting and inactivating *K*. *pneumoniae* that produces AmpC.

This study aimed to isolate and characterize the *Klebsiella pneumoniae* AmpC-specific phages from hospital wastewater.

**Methods:**

The hospital wastewater samples were collected from the sewage water effluent of a tertiary hospital at Universiti Sains Malaysia, located on the east coast of Malaysia. These samples underwent serial filtration and centrifugation processes for phage recovery. The phage solutions were undergoing a screening test by spot assay using clinical isolates of *Klebsiella pneumoniae* AmpC strain as amplification hosts. The isolated AmpC-phages were further studied and characterised to determine the phage’s host range, temperature, pH, and chloroform stabilities. High-Resolution Transmission Electron Microscopy (HRTEM) was performed to determine the phage type.

**Results:**

Thirty HWW samples were analyzed using four K. pneumoniae AmpC strains resulting in a total of 120 screening plates. The AmpC—Klebsiella pneumoniae (AmpC-KP) phages were detected in 31.70% (38/120) of the plates. The AmpC-KP phages had lytic diameters ranging from 1–3 mm, and a phage titer ranged from4×10^3^–3.2×10^7^ PFU/ml. The phages had a narrow–host range stable at a temperature range from -20–50˚C. The phages were also stable at pH ranging from 4 to 9 and at different concentrations of chloroform (5%,10%). Based on HRTEM, *Siphoviridea* was identified.

**Conclusions:**

The AmpC-phages were abundant in hospital wastewater, and HWW was a good source for AmpC-KP phages. The isolated AmpC phages had a high effectivity and specificity for AmpC-KP with a narrow host range and could survive under harsh conditions such as (temperature, pH, and chloroform).

## Introduction

Bacteriophages are viruses that can replicate specifically within bacterial cells and can be identified in practically all environments where live bacteria are present. The environments inhabited by bacterial hosts, including soil, sewage, and animal excretions, provide as abundance sources of various phages, providing the potential for bacteriophages isolation for therapeutic applications. Currently, a significant medical and societal issue is due to growing concern of Amp β lactamase–producing *Klebsiella pneumonia* [1].

AmpC enzymes are classified as Class C in the Ambler structural classification of β-lactamases [2]. However, they are classified as Group 1 in the Bush functional classification scheme [3]. The resistance genes encoded AmpC β-lactamases enzyme and led to antibiotics resistance were plasmid mediated and transferable to other bacteria [4]. AmpC β-lactamases *K*.*pneumoniae* (AmpC-KP) infections are a growing worldwide concern for humans [5]. subclinical AmpC-KP infection is associated with cardiovascular and inflammatory bowel disorders [6]. The most threatening species is AmpC-KP, which develops hyper-virulent colonies with increased virulence factors [7]. Additionally, the overuse and misuse of antibiotics lead to the development of antibiotic resistance [8]. So, antibiotic resistance development in KP increases the possibility of treatment failure [5]. Because of that, the researchers focused on developing alternative treatments for antibiotics by employing bacteriophages for the treatment of AmpC -KP infections [9].

Bacteriophages (phages) are viruses that specifically infect and replicate within bacterial cells. They are widely distributed across various environments and are considered the most abundant biological entities on Earth [10]. With an estimated global population ranging from 10^31^ to 10^32^ [11]. Hospital wastewater (HWW) is a significant reservoir for bacteriophages because HWW is considered a valuable source of bacteria and organic material [12]. The phages exhibit significant variations in their size, morphology, physiochemical properties and distribution of genetic material [10]. Phage treatment can potentially be effective against AmpC -KP infections [13], this study comprehensively focused on isolating characterizing, and identification of AmpC -KP bacteriophages from hospital wastewater using clinical isolates as the propagating host.

## Material and methods

### The study setting and design

This study was conducted at the Universiti Sains Malaysia (USM) Hospital, located on the east coast of Malaysia in Kelantan. Hospital wastewater (HWW) samples were collected from May to August 2023, with prior approval from the Asset Development and Management Department.

### Hospital wastewater samples collection

Thirty hospital wastewater (HWW) samples were manually collected from the effluent of Hospital Sains Malaysia at 10 a.m. using one-liter plastic bottles. The samples were stored at 4°C until analysis.

### Host strains

This study used four AmpC -KP (AmpC 152, AmpC 283, AmpC 177, and AmpC 297) clinical isolates obtained from clinical specimens in the Medical Microbiology & Parasitology laboratory, Universiti Sains Malaysia (USM), Malaysia. They were used as host cells to isolate and propagate the bacteriophages. Clinical isolates were stored at -80°C with 20% glycerol until further analysis was performed.

### Recovery of AmpC -*Klebsiella pneumoniae* phages from hospital wastewater

Thirty HWW samples undergone the recovery processes method based on previous published protocols with some modifications [14]. After collection, the HWW sample was kept in a cooled room at 4˚C for sediment to settle. Subsequently, the sample was filtered using a filter paper with a pore size of 11 μm. The filtrated sample was passed through 0.45 μm syringe filter paper was used to eliminate the bacterial debris and organic materials.

**Fig 1:**
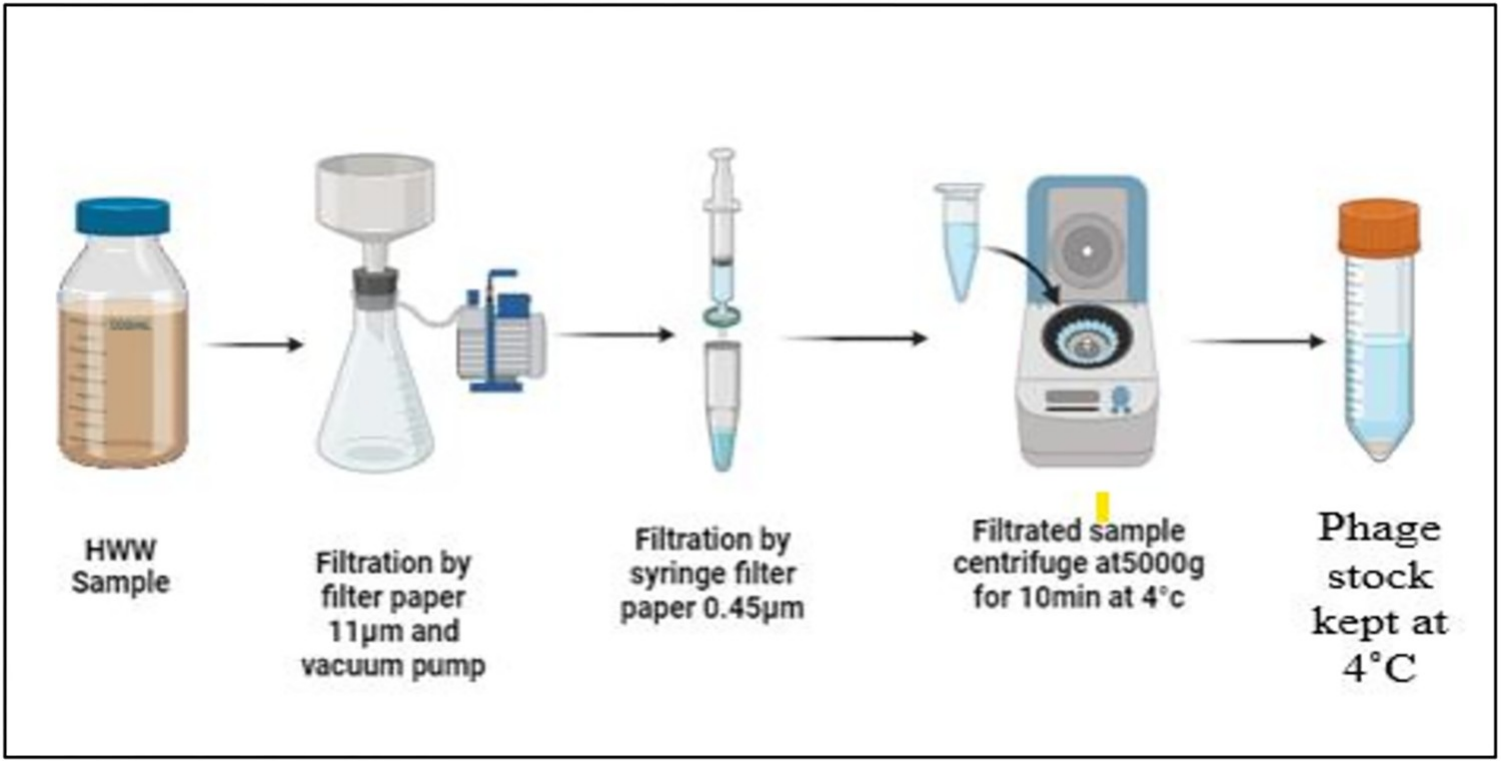
The diagram demonstrates the process for the recovery of bacteriophages from HWW samples.

The filtrated samples were centrifuged at 5000 g for 10 minutes at 4°C to concentrate the phages in the sample. After centrifugation, the phage stock was collected and transferred to a 50 ml conical tube and was kept at 4˚C for further analysis. Summary of phage recovery is “Fig 1”.

### Screening of AmpC -*Klebsiella pneumoniae* specific phages

**Fig 2:**
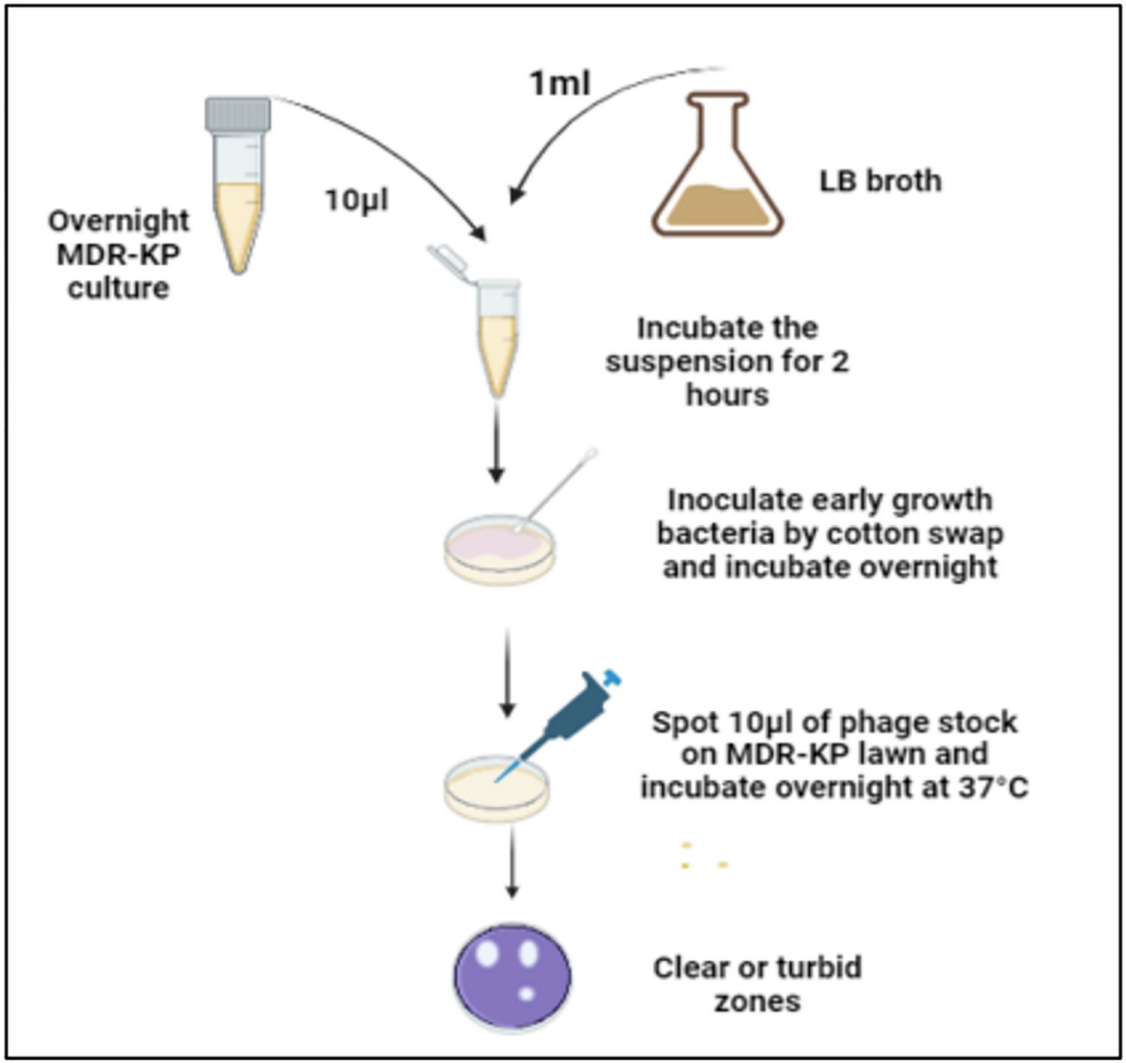
The diagram demonstrates the screening lytic phages against AmpC-KP strains in HWW samples by spot assay.

The spot assay method was used to assess the activity of phages against AmpC -KP. The procedures were conducted based on [1]. Thirty HWW phage stocks were analyzed to detect the AmpC-specific phages. The AmpC-KP strains stocks were thawed at ambient temperature. Afterward, the AmpC-KP overnight culture was obtained by incubating 10 μl of AmpC-KP strain stock in 1 ml of LB broth overnight. After incubation, the solution was homogenized by shaking the overnight AmpC-KP culture in a vortex mixer. Then, 10 μl of overnight AmpC-KP was added with 1 ml of LB broth and incubated for 2 hours until it reached early log-phase growth. Subsequently, a sterile cotton swab was used to swab each sample of AmpC onto nutrient agar, forming a bacterial lawn. Then, 10μl of phage stock was spotted on the bacterial lawn and allowed to dry at room temperature for 15 minutes, then incubated at 37˚C for 12 hours. The presence of lytic phages was determined by observing clear or turbid zones on the plate “Fig 2”. The test was performed with negative control (AmpC-KP bacterium monolayer without phage) and duplicates. The results were reported as either positive (+) or negative (-) for AmpC-KP samples.

### Enrichment of AmpC- specific phages

After screening the AmpC-specific phages, AmpC -KP phage stocks had undergone an enrichment process based on [15]. A 20μl mid-phase growth of AmpC-KP was added into 15 ml of Luria- Bertani (LB) broth with 15 ml of phage stock, 200 μl of 10 mM MgCl_2_, and 200 μl of 3 mM CaCl_2_. The mixture was incubated at 37˚C for 18 hours. After incubation, the mixture was centrifuged at 5000g for 10min at 4 ˚C. The supernatant was filtered using 0.22 μm Syringe filter. The phage suspension was collected and kept at 4˚C for further analysis.

### Isolation of AmpC- specific phages

The plaque assay method was applied to isolate AmpC-specific phages by using AmpC -KP strain as host cell based on [14]. The AmpC-KP strain stock was thawed at ambient temperature. Afterward, the AmpC-KP overnight culture was obtained by incubating 10 μl of AmpC-KP strain stock in 1 ml of LB broth overnight. The solution was homogenized by shaking the overnight AmpC-KP culture in a vortex mixer following incubation. Subsequently, a volume of 10 μl of an overnight culture of AmpC-KP was added to 1 ml of LB broth and subjected to incubation at a temperature of 37˚C for 2 hours. This process aimed to achieve the early log-phase growth of the AmpC-KP strain. The phage suspensions were diluted 10 -fold dilution from 10^−1^ to 10^−5^. Then, put 100 μl of diluted samples separately in equal volumes of early-phase AmpC-KP with 200 μl of 10 mM MgCl_2_ and 200 μl of 3 mM CaCl_2_. And place these solutions in a shaking incubator at 37˚C for 15 min. After that, add 3 ml of 3% soft agar to the solution with soft mixing. Then, pour the solutions with soft agar on nutrient agar plates. After pouring, incubate the plates at 37˚C overnight. After incubation, clear plaques appear. Then, pick the plaques of similar size and morphology. Afterward, place the isolated plaques in 1 ml of LB broth. Three repetitions were conducted for each plaque, using identical morphology, to confirm the individual isolation of phages. The negative control (monolayer of AmpC) and duplicates were analyzed. The isolated AmpC-specific phages (phage lysate) are stored at 4˚C for further analysis.

### Characterization of AmpC-specific phages

#### AmpC-specific phage titer and phage size

The plaque assay method was applied to determine the AmpC phage titer based on [14]. The negative control (monolayer of AmpC) and duplicates were analyzed. The negative control (monolayer of AmpC) and duplicates were analyzed. The results were presented as plaque-forming units per milliliter (PFU/ml) for phage titer and millimeters for phage size.

#### AmpC -specific phage host range

Six AmpC-specific phages (P1, P2, P3, P4, P5, and P6) were analyzed with other Gram-positive (*S*. *aureus* & *E*. *faecalis*) and Gram-negative (*E*. *coli* & *P*. *aeruginosa*) bacteria to assess the host range for AmpC phages using the spot assay method based on [13]. These phages were selected depending on high phage titer, and clear zone based on [16]. The negative control (monolayer of AmpC) and duplicates were analyzed. The results were reported as positive (+) or negative (-).

*Effect of temperature variation*. Six AmpC-specific phages (P1, P2, P3, P4, P5, and P6) with phage titer (10^7^ PFU/ml) were assessed for the activity of phages at different Temperatures, based on [17]. These phages were selected depending on high phage titer, clear zone, and host range [16]. All six specific phages were assessed at temperature values (-20, 4, 25, 50, and 90˚ C). 100 μl of AmpC-specific phage suspensions separately on equal volumes of LB broth and incubate at different temperatures (-20, 4, 25, 50, 90 ˚C) for 60 min. after incubation, the double-layer agar was applied. The negative control (monolayer of AmpC) and duplicates were analyzed.

*Effect of pH*. Six AmpC-specific phages (P1, P2, P3, P4, P5, and P6) with phage titer (10^7^ PFU/ml) were assessed for the activity of phages at variation pH based on [18]. These phages were selected depending on high phage titer, clear zone, and host range [16]. AmpC-KP specific phages were assessed at pH values of (2, 4, 7, 9, and 12). 100 μl of AmpC-specific phage suspensions were placed separately on equal volumes of adjusted LB broth at pH values (2, 4, 7, 9, and 12). The double-layer agar was applied. The negative control (monolayer of AmpC) and duplicates were analyzed.

*Effect of Chloroform*. Six AmpC-specific phages (P1, P2, P3, P4, P5, and P6) with phage titer (10^7^ PFU/ml) were assessed for the activity of phages at chloroform concentrations based on [19]. These phages were selected depending on high phage titer, clear zone, and host range [16]. The experimental phages were assessed at chloroform concentrations of 0%, 5%, and 10%. A volume of 100 μL of AmpC-specific phage suspensions was placed separately into equal volumes of adjusted LB.broth at different chloroform concentrations (0%, 5%, 10%). Afterward, the double-layer agar was applied. The negative control (monolayer of AmpC) and duplicates were analyzed.

### Identification of AmpC -specific phage morphology by High-Resolution Transmission Electron Microscope (HRTEM)

One AmpC-specific phage (P4) was identified under HRTEM (JEOL JEM 2100F FETEM) by using the negative stain method based on [20]. The P4 phage was selected depending on high phage titer, clear zone, and host range [16]. 3 μl of AmpC-specific phage was dropped on the parafilm. Then, the carbon-coated formvar grid was placed on the droplet sample for 5 minutes. After that, the grid was dried with filter paper. Then, it was placed in 3% uranyl acetate to stain phage for 3 minutes was then dried with filter paper. After that, the sample was viewed under HRTEM at 2500–30000 x and the voltage at 200 kV.

### Statistical analysis

The prevalence of AmpC–specific phages was expressed as frequency (n) and percentage (%), and the differences were determined by Kruskal- Wallis’s test and Friedman Test. The variables (dilution factor (10^−1^, 10^−2^, 10^−3^, 10^−4^, and 10^−5^), temperature values (-20, 4, 25, 50, and 90˚C), pH values (2, 4, 7, 9, and 12), and chloroform concentrations (0%, 5%, and 10%). The significance level was set at a P-value <0.05. All data analysis was performed using SPSS 28.0.

## Results

### Screening of AmpC-specific phages

**Fig 3:**
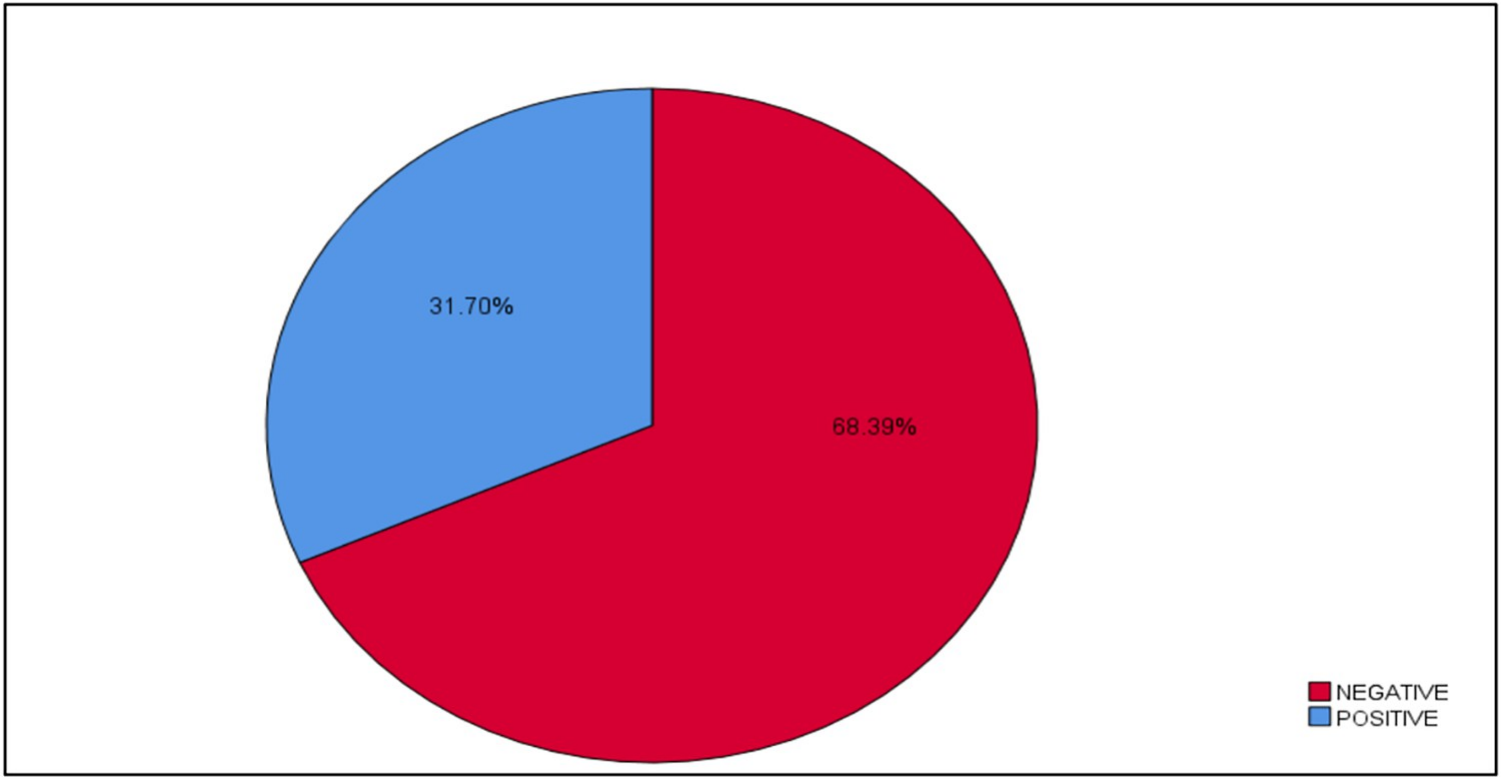
The prevalence of AmpC phages isolated from HWW by spot test.

**Fig 4:**
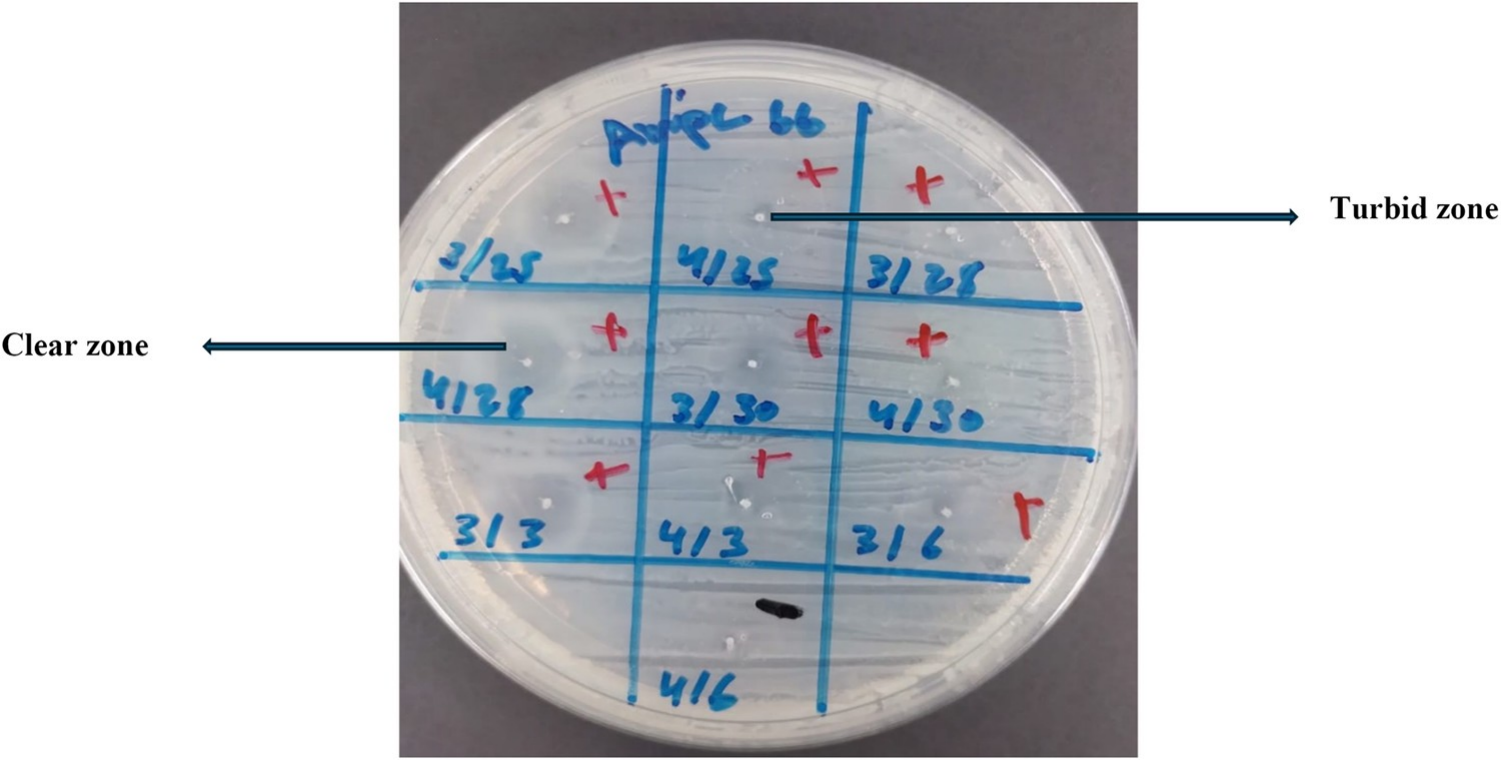
The AmpC-specific phages lytic zones formation: The clear lytic zone and turbid lytic zone.

A total of thirty HWW samples were obtained and analyzed, and four samples of AmpC-KP were utilized in the screening using the spot assay method. The prevalence of AmpC phages was 31.70% (38/120) [0.234,0.400, 95% CI] “Fig 3”. The zones obtained from the spot assay were clear and turbid zones “Fig 4”.

### Characterization of isolated AmpC- *K*. *pneumoniae*-specific phages

#### Determination of the phage titer and phage size

**Fig 5:**
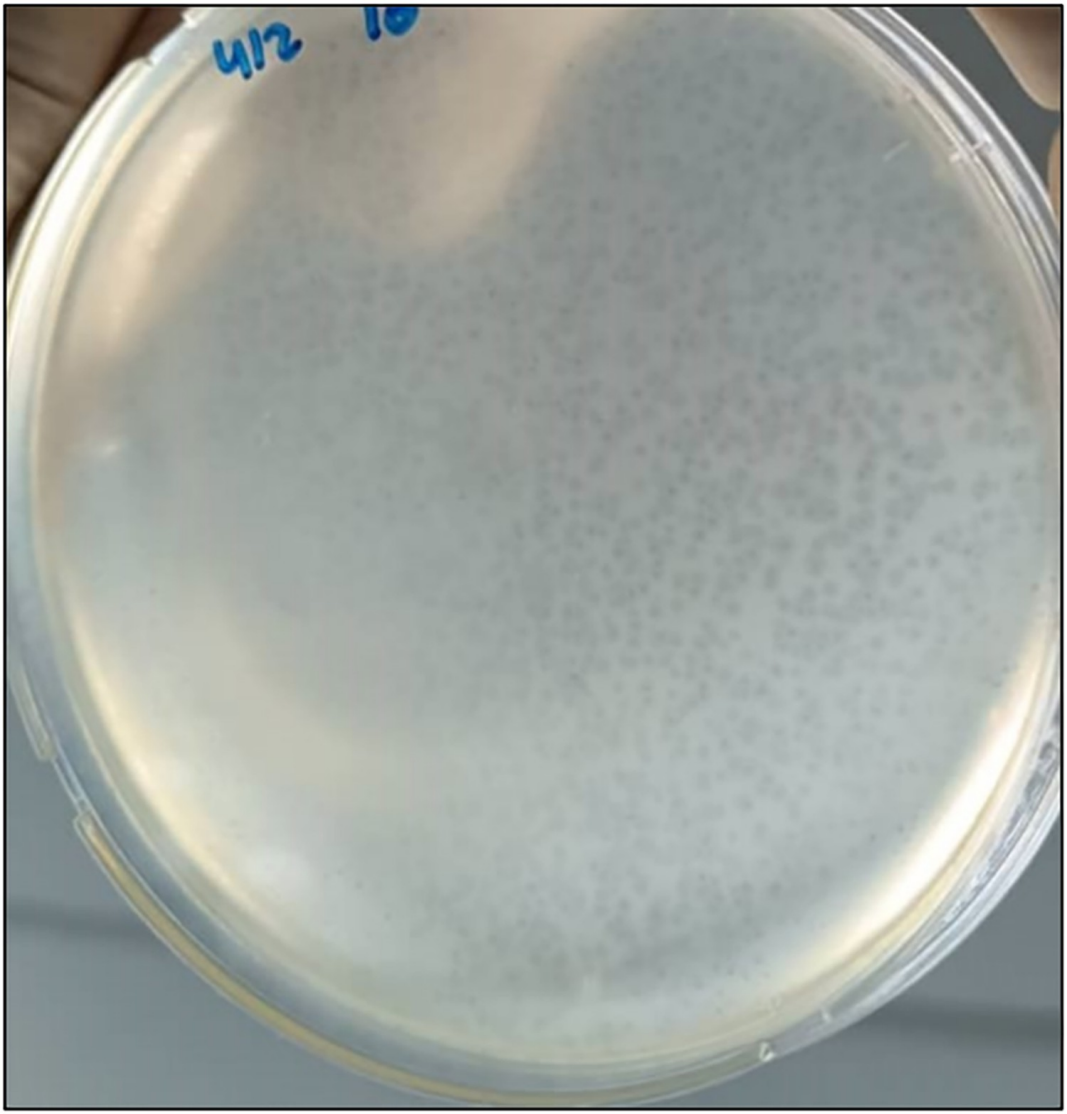
The plaque number was TNTC (>300 plaques) using the plaque assay method.

**Fig 6:**
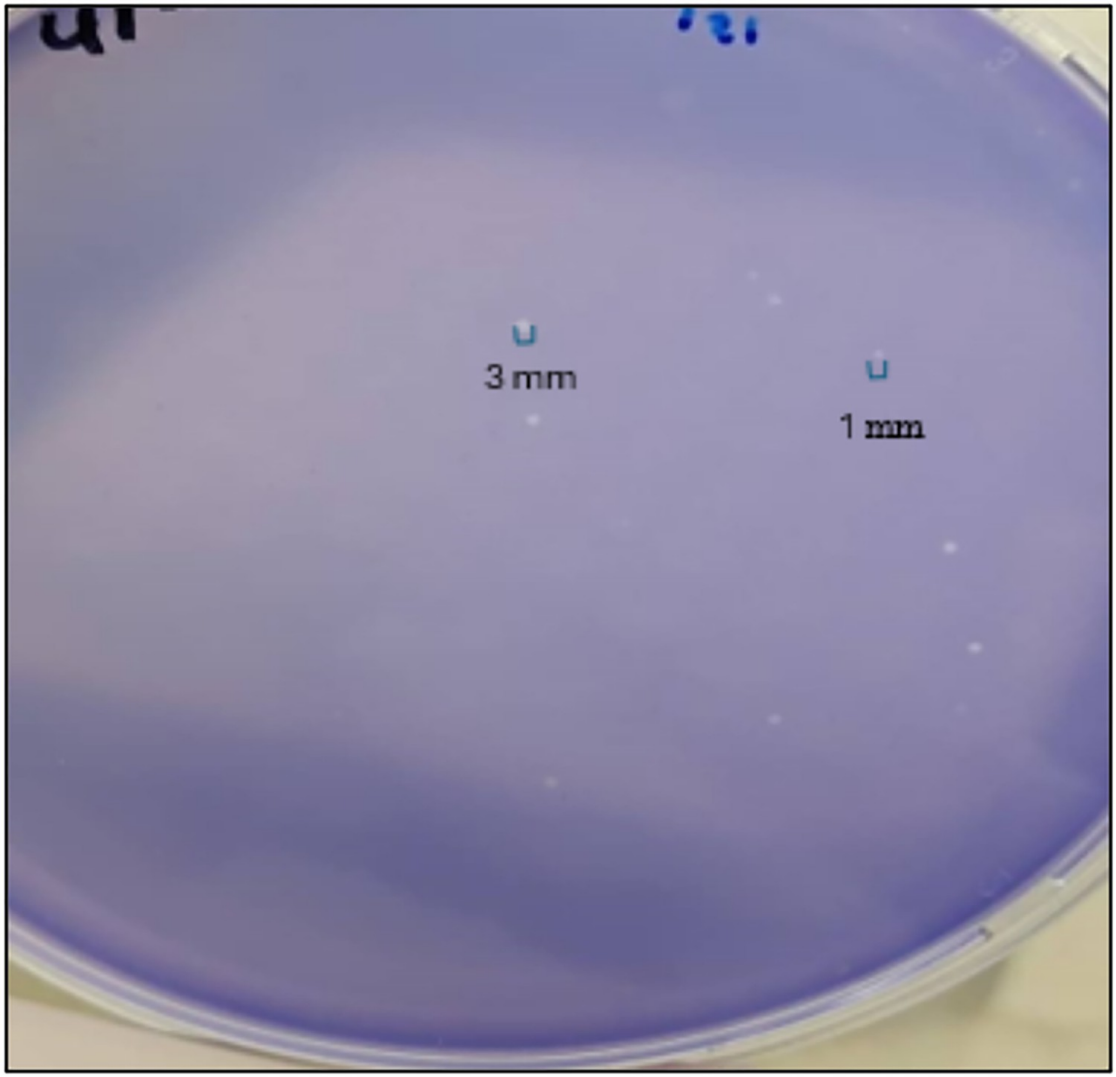
The AmpC-KP phage size ranges from 1 to 3mm.

The phage titer was measured by counting the plaques (PFU/ml) using plaque assay. The plaque number range (0–300 plaques). And the “Too Numerous to Count” (TNTC) was also counted. The term "TNTC" describes the number of plaques exceeding 300 plaques, "Fig 5”. The AmpC phages have a titer range (4×10^3^–3.2×10^7^) PFU/ml. The dilution exhibited a statistically significant (*P*-value <0.05) relationship with phage titer “Table 1”. The phage size was presented as diameter in (mm). All AmpC- specific phages have a diameter range from 1 to 3 mm, “Fig 6”.

**Table 1:** The comparison between the dilution with AmpC-KP phage titer.

** **	**10^-1**	**10^-2**	**10^-3**	**10^-4**	**10^-5**	**X2**	***p*- value#**
	**Median**	**Median**	**Median**	**Median**	**Median**	**(df)**	
	**(IQR)**	**(IQR)**	**(IQR)**	**(IQR))**	**(IQR)**		
						16.488	0.002
**Phage titer (PFU/ml)**	2.4×106	2.1×106	1.2×106	3.0×105	1.1×105		
	(4.9×107)	(1.2×107)	(3.2×107)	(1.0×106)	(3.4×105)		

# Kruskal-Wallis Test was used

* Significant P <0.05.

#### Determination of the AmpC- phage host range

The host range of AmpC-KP phages was determined using the spot assay method; two Gram-negative bacteria (*E*. *coli* & *P*. *aeruginosa*) and two Gram-positive bacteria (*S*. *aureus* & *E*. *faecalis*) were used. Following the incubation process of phages with bacterial isolates were not noticeable against the other strains from both Gram-positive and Gram-negative bacteria “Table 2”.

**Table 2:** The host range of AmpC- specific phages by spot assay.

AmpC—phages	*E*. *coli*	*P*. *aeruginosa*	*S*. *aureus*	*E*. *faecalis*
**Phage 1**	-	-	-	-
**Phage 2**	-	-	-	-
**Phage 3**	-	-	-	-
**Phage 4**	-	-	-	-
**Phage 5**	-	-	-	-
**Phage 6**	-	-	-	-

#### AmpC- *K*. *pneumoniae* phages thermal stability

**Fig 7:**
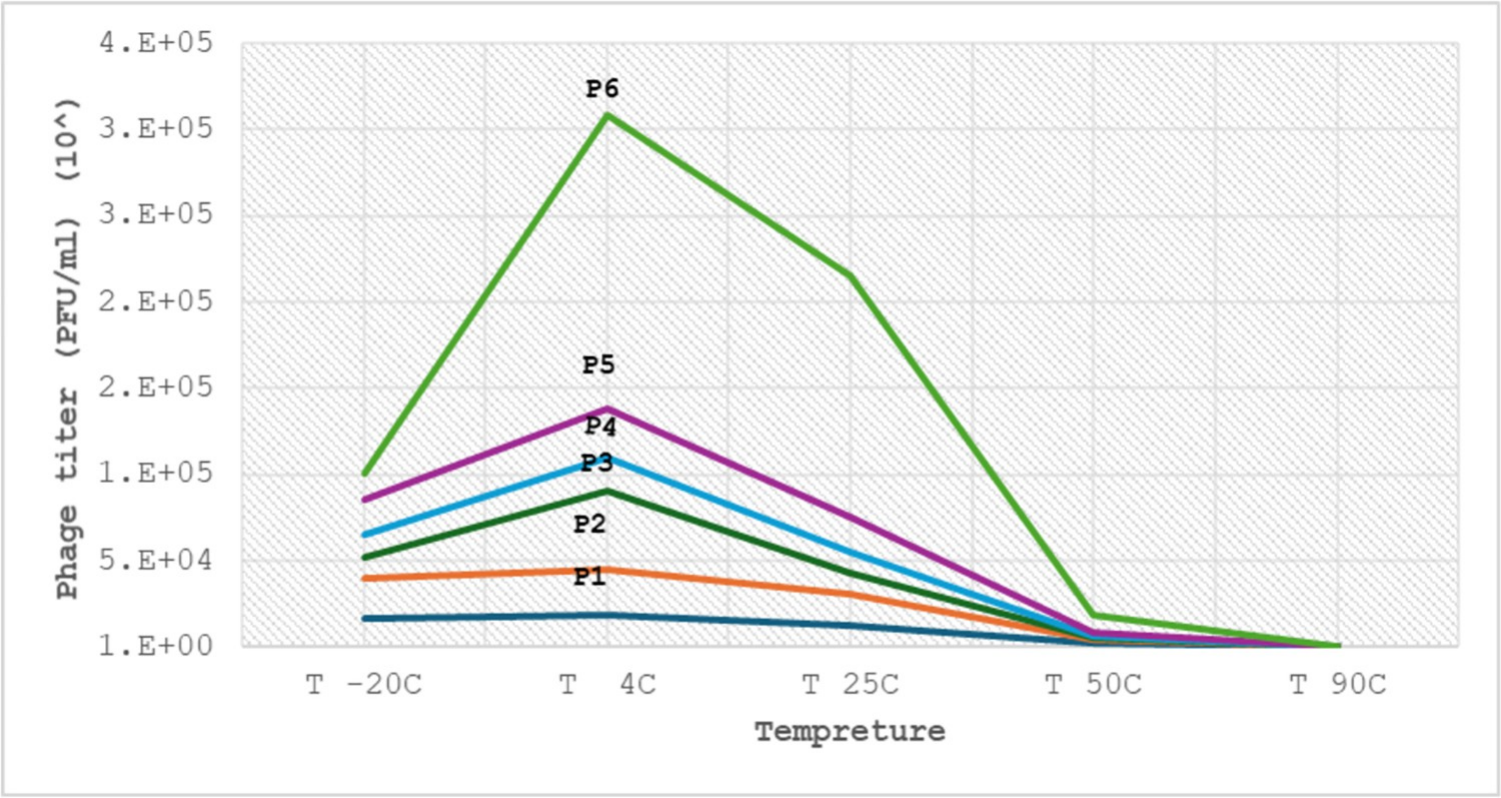
AmpC phages stability at different temperatures by plaque assay.

Six isolated AmpC-KP phages were analyzed to detect effective temperatures from -20 to 90˚C on the original AmpC phage titer (10^7^) PFU/ml. All phages showed stability between -20 to 50˚C. The optimal temperature for the stability of phage growth was 4˚C. The phage titer observed a small decline at temperatures of –20˚C and 50˚C. At 90˚C, the phage titer reached zero, “Fig 7”. The temperature values exhibited a significant effect (P < 0.05) on the phage titer for all investigated phages, “Table 3”.

**Table 3:** The comparison between the temperature values with AmpC phage titer (PFU/ml).

	AmpC	
	X^2^	*p* -value
**Temperature-20°C**	23.322	<0.001*
**Temperature 4°C**		
**Temperature 25°C**		
**Temperature 50°C**		
**Temperature 90°C**		

# Friedman Test was utilized

* Significant P <0.05.

#### AmpC- *K*. *pneumoniae* phages pH stability

**Fig 8:**
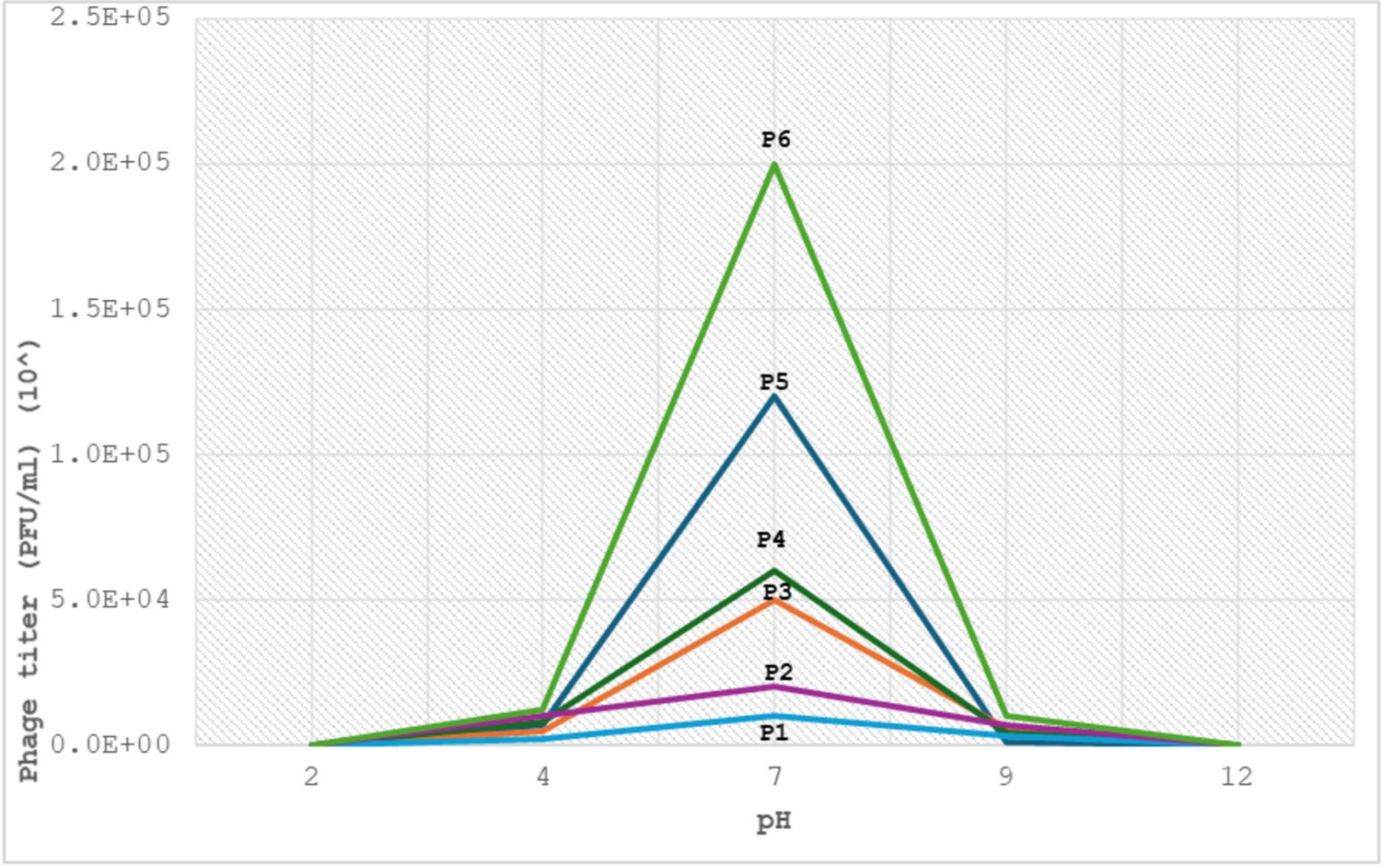
AmpC phages stability at different pH by plaque assay.

Six isolated specific AmpC phages were analyzed to assess the activity of phages at different values of pH from 2 to 12. All phages were stable at 4–9 pH. The optimal pH is 7. Meanwhile, all phage titers dropped when pH values were less than 4 and more than 9. On the other hand, all AmpC—phage titer reached zero when pH values were at 2 and 12 “Fig 8”. The pH values exhibited a significant effect (P < 0.05) on the phage titer for all tested phages, “Table 4”.

**Table 4:** The comparison between the pH values with AmpC phage titer (PFU/ml).

	AmpC	
	X2	*p* -value
**pH 2**	22.877	<0.001*
**pH 4**		
**pH 7**		
**pH 9**		
**pH 12**		

# Friedman Test was utilized

* Significant P <0.05.

#### AmpC- *K*. *pneumoniae* phages chloroform stability

**Fig 9:**
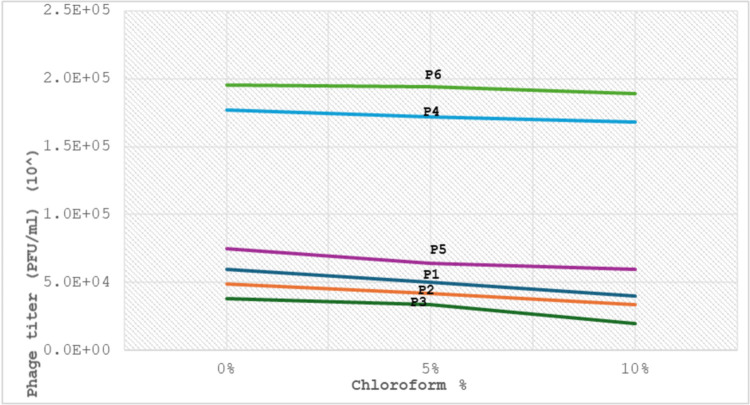
AmpC phages stability at different chloroform concentrations by plaque assay.

Six isolated specific AmpC phages were analyzed to assess the activity of phages at different concentrations of chloroform (0%, 5%, 10%) for 2-hour incubation. All phages showed optimal phage growth at 0% and 5% respectively. While all AmpC—phage titers slightly dropped at 10%, “Fig 9”. The chloroform concentrations exhibited a significant effect (P < 0.05) on the phage titer for all six isolated phages, “Table 5”.

**Table 5:** The comparison between the chloroform concentration with AmpC phage titer (PFU/ml).

	AmpC	
	X2	*p* -value
**Chloroform 0%**	12	<0.002
**Chloroform 5%**		
**Chloroform 10%**		

# Friedman Test was utilized, * Significant P <0.05.

### Morphological identification of AmpC- *K*. *pneumoniae* phage by High-Resolution Transmission Electron Microscope (HRTEM)

**Fig 10:**
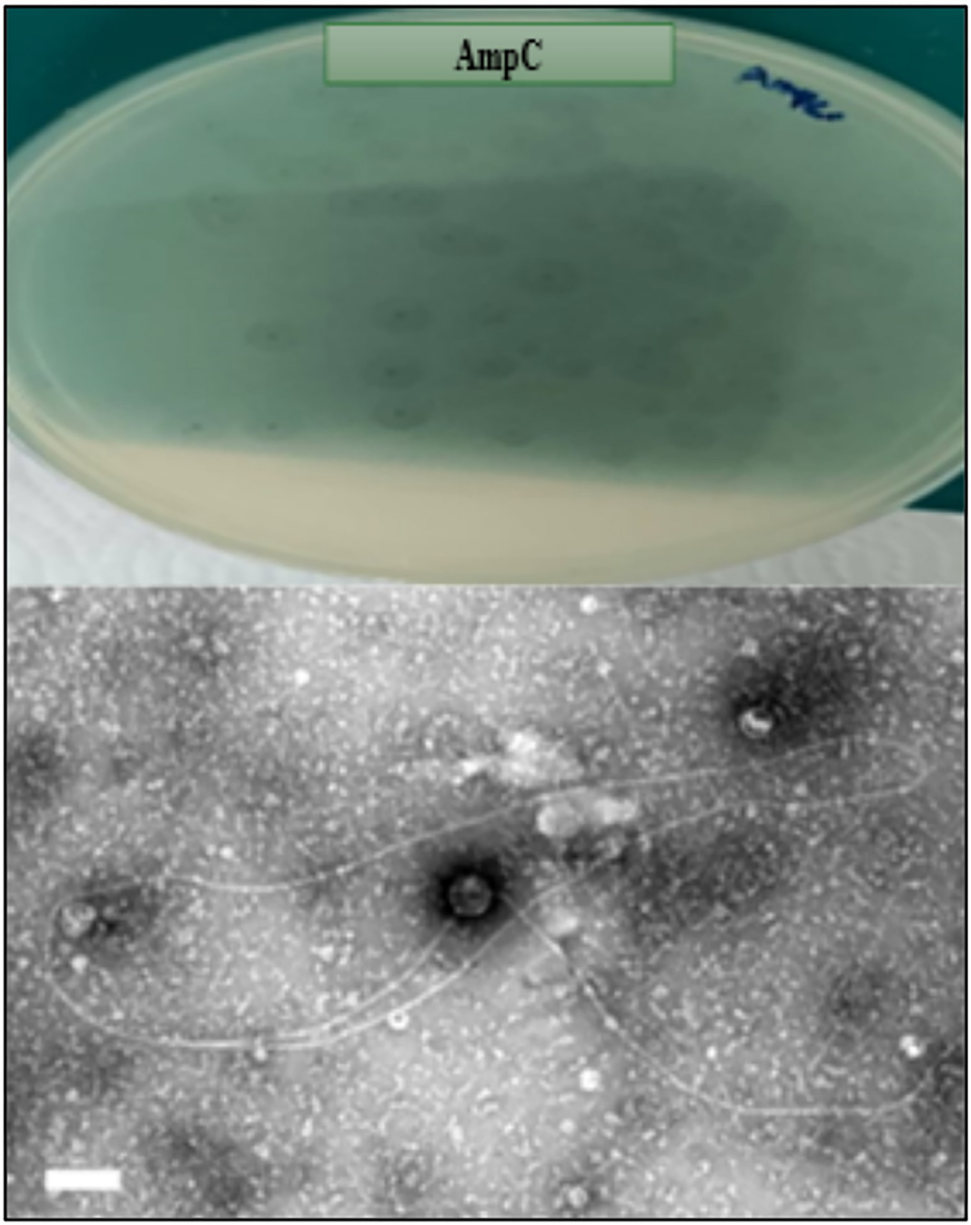
The morphology of AmpC phage (*Siphoviridae*) under HRTEM by negative staining.

One selected isolated AmpC-specific phage (P4) was identified under HRTEM using the negative stain method. The morphology of the AmpC phage was *siphoviridea*, which had a narrow, long tail and small head based on [21], “Fig 10”.

## Discussion

Multidrug-resistant AmpC-*K*. *pneumoniae* strains utilized as propagation host for bacteriophage isolation. AmpC-*K*. *pneumoniae* is a global concern, and resistance to broad-spectrum β-lactams caused by AmpC β-lactamase enzymes is becoming growing. Considering the Southeast Asia’s record for high antibiotic resistance incidences [22]. In Malaysia, the prevalence of AmpC -KP was observed 32.1% [23]. Bacteriophages are viruses that may infect and destroy bacteria, making them an effective strategy to eliminate antibiotic-resistant infections. Phage therapy has several advantages over antibiotic therapy, including high host-bacteria specificity, self-limiting properties, a relatively simple and low-cost preparation process, and high activity in inhibiting biofilm formation [24].

In this study we observed that the prevalence of AmpC bacteriophages in HWW was 31.70%. Our findings align with those of a recent study [25] which reported that the prevalence of bacteriophages in wastewater treatment facilities in Winnipeg, Manitoba, ranged from 20% to 49% [26]. On the other hand, a recent study [27] showed a higher prevalence of *K*.*pneumoniae* phages, at 63%. The main factor that influences the prevalence is the phage effectiveness, which depends on the phage titer, type of phage, concentration of bacteria, and physical condition such as (temperature, pH, and rain season) [28].

Our findings revealed that the AmpC phages have a phage titer range (4×103–3.2×10^7^) PFU/ml. So, this phage titer is effective in therapeutic applications, as therapeutic dosages frequently necessitate phage titers between 10^3^ and 10^9^ [29]. This finding is constant with [30] which showed similar findings. While a previous study [31] revealed the *E*. *coli* phage titer was 10^16^ PFU/ml. The bacteriophages abundance in HWW related to environmental conditions, divers microbial load, chemical component, organic material and suspended solid can be presented in HWW[32].

In the current study, the AmpC phages have a 1–3 mm plaque diameter range; according to these findings, these phages exhibit a significant impact on AmpC-KP, and these phages are suitable for therapeutic applications[33]. This finding is related with a recent study [34] the *K*. *pneumoniae* phage has a 2–3 mm plaque diameter range. Contrary to Ullah (2021), it was estimated that the *E*.*coli* phage has a 0.5–1 mm plaque diameter range [35]. The diversity in plaque diameter related to virulent phage, phage size, concentration of soft agar [36], and Ca^+2^ and Mg^+2^ concentartion [37]. Additionally, the bacterial load, chemical and organic component can be presented HWW [14].

All six isolated AmpC phages showed high specificity for the AmpC strain and narrow host range, constant with recent studies [38,39] showed similar findings. The host range can be related to the bacterial load, chemical and organic component can be presented HWW [14].

In the current study, all six phages have a wide range of thermal stability from -20 to 50˚ C. The optimum temperature was at 4 ˚C. All AmpC isolated phages were inactive at 90˚ C. These findings related with [40] showed similar findings. Regarding these results, the variation in temperature values significantly affected AmpC phage titer (P <0.05). These findings related to a previous study [41] established that the temperature values significantly affected *E*.*coli* phage titer. The investigated AmpC isolated phages exhibit more efficacy against AmpC-*K*. *pneumoniae* at a wide range of temperatures from -20˚c to 50˚c; this finding has potential benefits for therapeutic applications. The AmpC-Kp phage titers were influnce by the variation tempreture values related to the type of phage and bacteria, bacterial and phage load in HWW [25].

All AmpC phages have a wide pH range from 4 to 9. The optimum pH was at 7 pH, while all phages were non survived at 2 and 12. These findings are constant with a recent study [42] that revealed similar findings. according to this result, the variation in pH values significantly affected the AmpC phage titer (P <0.05). This result is related to a recent study [43] revealed that the *K*.*pneumoniae* phage titer is significantly influenced by variation in pH values. Our findings show that all expermented isolated specific phages could be beneficial when utilized for therapeutic purposes, especially in oral therapy. The AmpC-Kp phage titers were influnce by the variation pH values related to the type of phage and bacteria, bacterial and phage load in HWW [44].

All six phages have a stability range from 5% to 10% for chloroform stability. According to our results, the variation in chloroform concentrations significantly affected AmpC phage titer (P <0.05) related to the previous study [45] that revealed similar results. This findings related to the prescence of protein capsid in bacteriophages structures [46]. Our phages exhibited higher tolerance to extreme, harsh circumstances, signifying increased efficacy for therapeutic applications.

The current study successfully identified an isolated AmpC phage utilizing morphological analysis and negative staining. The phage had features common to the *Siphoviridae* family, such as a narrow, long, contractile tail and a small head. Given the exceptionally high virulence and adsorption properties commonly associated with *Siphoviridae* phages [21], this phage has tremendous therapeutic promise, particularly in developing of phage cocktails to treat AmpC-KP infections.

## Conclusion

Bacteriophages are prevalent in the environment, particularly among HWW. The AmpC -KP caused of numerous infectious diseases. In the recent study, bacteriophages against AmpC-KP have been effectively isolated from HWW at Universiti Sains Malaysia in Kelantan, Malaysia.

Our phages showed a high level of efficacy against AmpC-KP strains demonstrated by the morphology of phages, and phage titer. these phages have a prevalence range in HWW (31.70%) with phage titer range (4×103–3.2×10^7^), and phage size range (1–3 mm). All AmpC phages are capable of surviving unfavorable conditions, including temperature, pH, and chloroform. The AmpC phagewas notably similar to *Siphoviridea* (filamentouse phage). Therefore, all investigated phages have the potential to be effective in the treatment of AmpC infections.
